# Notes and News

**Published:** 1899-07-08

**Authors:** 


### Notes and News.

(Collected and Communicated.)

The Prince and Princess of Wales will open the new buildings of the Alexandra Hospital for Children with Hip Disease, Queen Square, Bloomsbury, on Thursday, July 20th, at noon.

At a meeting of the medical profession in Birmingham a series of resolutions strongly condemnatory of the proposed consulting institution were passed unanimously. The reasons adduced in favour of this condemnation seem indisputable.

In answer to a question in the House of Commons last Friday, Mr. Chaplin said that of the 83 cases of small-pox at Hull as to which the Local Government Board had information, 62 showed evidence on examination of having been vaccinated, and 21 appeared to have been unvaccinated. The rate of death amongst the patients exhibiting evidence of vaccination had been 9·7 per cent., whilst amongst those exhibiting no evidence of vaccination 57·1 per cent.

In view of the probable influence of the sucking of lead pencils, which is so common a habit among school children, in spreading diphtheria and other infectious diseases, it has been proposed in America that pencils for use in schools—and why not in other places?—should be impregnated with some bad-tasting substance which might deter children—we might add other people as well—from putting pencils in their mouths. This is one of the dirtiest of habits, and one that is neither confined to children nor to the lower strata of society.

A large audience, including many ladies, assembled in the anatomical theatre at Charing Cross Medical School, on Wednesday afternoon, in order to witness the distribution of prizes to the students of the Medical School. In the absence of Lord James of Hereford from indisposition, the ceremony was performed by the Solicitor-General (Sir Robert Finlay). After he had duly presented the scholarships, medals, prizes, and certificates awarded during the summer session 1898 and the winter session 1898-99, Sir Robert delivered a charming little speech, in which he congratulated the successful students, and expressed his apprehension that Mr. B. A. Nicol would want a four-wheeler to take his prizes away.

On June 27th Professor Virchow formally opened the new pathological museum, which is to be called after him. It has been built under his superintendence, and contains a collection of more than 20,000 specimens wholly collected by Professor Virchow himself.

Although the prevalence of plague in India has lately diminished considerably it seems to be very bad again in Hong Kong. A telegram from the Governor of that colony, received at the Colonial Office on Monday, stated that there were 142 new cases of plague, and 144 deaths, in the colony last week.

Some time ago we gave an account of the Rev. Gilbert Reid's scheme to establish an international institute to disseminate knowledge at Pekin. Since then Mr. Reid has been working to effect his object, and has secured both encouragement and substantial monetary assistance in Holland, Denmark, Germany, and in Paris.

The trustees of the Women's Medical College of the New York Infirmary have issued a statement to the effect that there is no longer a valid reason for its continuance, seeing that the medical department of Cornell University is now open to women on the same terms as to men. The college will now, therefore, be closed after a career of thirty-five years.

It is amusing, in the face of the outcry frequently made by ill-informed persons for rate-supported hospitals, to read the report of a recent occurre nce brought before the Penzance Town Council. It appears that a scarlatina patient was received into the Infectious Diseases Hospital, and remained for eight weeks. At the end of the time the medical officer presented a bill of £32 for medical and nursing attendance and maintenance to the sanitary committee. An enquiry was made, and at the meeting it was not held necessary to go into details of the result, but the chairman of the committee stated they were wiser than six months before, and such an occurrence would not happen again. Although the extravagance in supplies was not fully gone into, it was mentioned that one item in the accounts was a bill for 27 dozen eggs.

Protests have often been raised against the risks which are run so recklessly by athletes in their struggle for "records," but it seems to us that the matter is brought to a climax in the recent cycling performance, when a man named Murphy—a man who now, we suppose, will be held to be a hero of first magnitude—rode a mile in 57 4-5 sec., being protected from the wind by a wind-shield carried by a railway engine. We read that "he finished wobbling in every direction, and collided with the car which he was following," and this at over 60 miles an hour! "The spectators and officials were in a most nervous state, realising that the slightest error meant death," as well they might be; and in the end "men standing on the platform of the car snatched him up from a most perilous position, and laid him on the platform only half conscious." This is modern athleticism! Next time any of us travel by one of our quickest expresses let us put our head out of the window and think what it would be like to pick up a cyclist running on a track beside! We take our report from the *Times*.

